# Knowledge, Attitudes and Practices of Intensive Care Unit Nurses Regarding Critical Care Ultrasound: A Cross‐Sectional Study in Southwestern China

**DOI:** 10.1111/nicc.70170

**Published:** 2025-09-11

**Authors:** Lin Yang, Lei Lei, Shuai Zhang, Xia Zhang, Min Xu

**Affiliations:** ^1^ Department of Pediatric Intensive Care Unit Nursing West China Second University Hospital Sichuan University Chengdu China; ^2^ Key Laboratory of Birth Defects and Related Diseases of Women and Children (Sichuan University) Ministry of Education Chengdu China; ^3^ West China School of Nursing Sichuan University Chengdu China

**Keywords:** attitudes, critical care ultrasound, ICU nurses, knowledge, practices

## Abstract

**Background:**

Critical care ultrasound is a promising technology for assessment and operational assistance in critical care. However, research on its application in critical nursing remains limited in China.

**Aim:**

Our aim was to evaluate the knowledge, attitudes and practices of intensive care unit (ICU) nurses regarding critical care ultrasound.

**Study Design:**

A cross‐sectional, questionnaire‐based study. Data were collected via an online survey from April 1 to April 30, 2023, across six hospitals in Southwestern China. A structured questionnaire on critical care ultrasound was administered to 404 ICU nurses. Scores were converted to a percentage scale, with ≤ 60% considered negative, < 80% intermediate and ≥ 80% excellent.

**Results:**

Overall, 64.4% (260/404) of nurses scored at an intermediate level. In the knowledge dimension, 70.8% (286/404) of nurses had negative scores; in the attitude dimension, 49.0% (198/404) of nurses had an intermediate attitude; in the practice dimension, 22.8% (92/404) of nurses struggled to effectively implement clinical practice. Univariate analysis revealed that nursing object (*F* = 11.520, *p <* 0.01), ICU classification (*F* = 3.613, *p* = 0.029) and education level (*F* = 4.765, *p* = 0.010) significantly influenced the scores of nurses' knowledge, attitudes and practices regarding critical care ultrasound. Multivariate analysis revealed surgical ICU classification significantly predicted lower scores versus general ICU in total scores (*β* = −0.189, 95% CI [−29.499, −4.850], *p* < 0.01). Paediatric cases showed reduced scores versus adults in total scores (*β* = −0.233, 95% CI [−12.638, −1.989], *p* < 0.01). Conversely, mixed adult–paediatric care demonstrated superior total scores (*β* = 0.182, 95% CI [0.710, 15.448], *p* < 0.01) versus adult‐only care.

**Conclusions:**

ICU nurses hold a positive attitude toward critical care ultrasound technology, generally believing that it can assist their work and improve patient outcomes. However, the current practical application of critical care ultrasound by ICU nurses is insufficient, particularly due to a lack of theoretical knowledge related to the technology.

**Relevance to Clinical Practice:**

These findings can assist nursing management in effectively developing future training programmes for critical care ultrasound, promoting its application and development in ICU nursing practice.

**Impact Statements**

- What is known about the topic
  ○ The nurse‐led application of critical care ultrasound can remarkably improve nursing quality and patients' outcomes.
  ○ There are few reports on the application of critical care ultrasound in the nursing field in China, and the published literature on critical care ultrasound nursing mostly focuses on the application effects of ultrasound technology.
- What this paper adds
  ○ To our knowledge, this study investigated knowledge, attitudes and practices toward critical care ultrasound among nurses in China for the first time.
  ○ More than 70% of the nurses scored poorly in the part of critical care ultrasound knowledge. This study revealed significant gaps in ICU nurses' knowledge and practices regarding critical care ultrasound technology, despite generally positive attitudes.

## 1 Introduction

As a bedside real‐time and non‐invasive imaging examination, critical care ultrasound (CCUS) can rapidly and comprehensively visualise and assess the condition of critically ill patients, which helps to improve the accuracy of clinical decision‐making. Nowadays, the application of CCUS in the nursing field is becoming increasingly widespread and has the potential to become a powerful assistant for nurses when assessing and operating on critically ill patients. Currently, CCUS can assist nurses in screening for deep vein thrombosis and evaluating patients' gastrointestinal function, lung function, pressure injury status, skeletal muscle function and so on [1, 2]. Meanwhile, it also plays a crucial role in various ultrasound‐guided catheterisation procedures [3]. With increasing adoption, CCUS is progressively transforming critical care nursing workflows by enabling independent real‐time bedside evaluations and reducing reliance on radiological examinations, thereby optimising clinical processes [4, 5].

## 2 Background

The advantages of CCUS have been validated by numerous domestic and international studies. During the Coronavirus Disease 2019 pandemic, critical care nurses can effectively evaluate the effectiveness of chest physiotherapy by conducting ultrasound‐guided lung examinations [6]. Steinwandel reported that nurses in haemodialysis units can use CCUS to evaluate the inferior vena cava, which helps to determine the ultrafiltration target of haemodialysis and reduce the risk of adverse complications such as hypotension [7]. Palese found that ultrasound examination can measure the post‐void residual urine volume and monitor the bladder volume in real‐time. This is helpful in reducing the number of urinary catheters used and the duration of their use, thus decreasing catheter‐related urinary tract infections [8]. In the paediatric intensive care unit (PICU), CCUS can significantly improve the prognosis of children with profound dengue shock syndrome and undergoing mechanical ventilation by monitoring their haemodynamics and fluid administration [9].

Although numerous domestic and international studies have demonstrated that ultrasound‐assisted nursing assessments can improve patient outcomes and enhance care quality, the current application of ultrasound in nursing practice remains limited. Evidence from domestic and international studies on critical care ultrasound applications demonstrates that nursing utilisation remains predominantly focused on ultrasound‐guided catheter placement procedures, including peripherally inserted central catheters (PICCs), nasoenteric tubes and urinary catheters, with particular efficacy in patients with difficult venous access [10, 11, 12]. Research on the application of CCUS in nursing assessment remains limited, with particularly scarce data documenting its real‐world implementation in ICU clinical settings. Multiple studies have shown that the main obstacles to the application of ultrasound technology in the nursing field include a shortage of nurses with ultrasound qualifications, insufficient dedicated equipment and a lack of a standardised training system [13, 14, 15, 16].

With the rapid development of critical care medicine, guidelines and expert consensuses for CCUS have been introduced successively around the world [17, 18, 19]. Meanwhile, quite a number of studies are being conducted to establish a training system for critical care ultrasound nurses [20]. These efforts have laid a theoretical foundation for performing ultrasound operations and cultivating professionals. However, currently in China, there are no specialised certification and systematic training for ultrasound nurses, and there are various difficulties in the clinical application of ultrasound [21]. Similar situations are reported in other literature, pointing out the lack of specific training pathways and non‐homogeneous programmes [22]. One qualitative study shows that CCUS nurses face challenges such as difficulty in obtaining standard images, interpreting images and ensuring the standardisation and accuracy of clinical practice [23]. These practical difficulties hinder the large‐scale clinical application of critical care ultrasound technology.

Undoubtedly, CCUS is one of the most promising assessment and operation‐assisting technologies at present. However, there are few reports on the application of CCUS in the nursing field in China. The existing literature on CCUS nursing primarily focuses on two aspects: documenting the clinical outcomes of ultrasound technology implementation and reporting on the development of critical care ultrasound training systems [24, 25]. Currently, there is no available data documenting the proportion of nurses in China who have mastered CCUS techniques or those who routinely apply this technology in clinical practice. A CiteSpace‐based visualisation analysis of ultrasound technology in Chinese critical care nursing revealed that CCUS remains a relatively unfamiliar and technically challenging procedure for the vast majority of clinical nurses [26].

## 3 Aim

Therefore, the purpose of this study is to investigate nurses' knowledge, attitudes and practices regarding ultrasound by leveraging the knowledge–attitude–practice (KAP) model. The primary goal is to provide a theoretical basis for conducting targeted training of CCUS and to analyse the possible difficult factors in the application of CCUS, thus laying the foundation for ultimately promoting the large‐scale application of CCUS in clinical practice.

## 4 Design and Methods

### 4.1 Study Setting and Sample

A quantitative cross‐sectional survey was conducted in this study by employing convenience sampling. The data were collected from nurses who were employed in the ICUs of six hospitals in Chengdu City, Sichuan Province, China, within the period from 1 April 2023 to 30 April 2023. The sample size was calculated based on the Kendell principle prior to the commencement of the study, and it was calculated to be within a range of 10–15 times the number of questionnaire items. The total number of questionnaire items was 36. Considering the anticipated follow‐up loss rate of 10%–20%, a minimum required sample size of at least 396 participants was determined.

All participants were required to fulfil the following inclusion criteria: had (a) obtained a Nurse Practising Qualification Certificate; (b) were currently working in the ICU for 1 years or more and (c) volunteered to participate in the study. The exclusion criteria encompassed continuing education nurses, interns and nursing students. All nurses who met the eligibility requirements completed the survey using an online questionnaire accessible through https://jsj.top/f/eeOycE. This study followed the Strengthening the Reporting of Observational Studies in Epidemiology (STROBE) guidelines for cross‐sectional studies.

### 4.2 Data Collection Tools and Methods

The questionnaire used in this study consisted of two parts. (1) General data: customised by researchers, including gender (female/male), age, education level (diploma/bachelor's degree/master's degree or above), ICU experience (less than 5 years/5–10 years/11–15 years/16–20 years/more than 20 years), professional title (primary nurse/intermediate or supervisor nurse senior), ICU classification (internal medicine ICU/surgical ICU/general ICU) and nursing population (adult/children/mixed adult and children). (2) ‘The knowledge, attitudes and practices assessment scale of ultrasound for ICU nurses’: this scale was designed by the investigator according to the purpose of the study. We conducted two rounds of Delphi expert consultations on the basis of extensive literature review. Experts were selected based on their demonstrated expertise in CCUS clinical applications, as verified by formal certification in CCUS training, along with a minimum of 8 years of ICU clinical experience, while excluding any participants with potential conflicts of interest related to this study. The expert panel comprised nine specialists in critical care medicine and nursing (including two full senior, three associate senior and four intermediate title holders; three PhD, four master's and two bachelor's degree holders; four physicians and five nurses, all with ≥ 10 years' ICU experience). The expert authority coefficients (Cr) for the two rounds were 0.816 and 0.869 respectively, indicating high reliability. The pretest data showed that the total Cronbach's α coefficient of this scale was 0.895, confirming good internal consistency.

The second part of the questionnaire consisted of 29 questions related to knowledge, attitudes and practices regarding ultrasound. The knowledge section consists of 7 items, the attitudes section is composed of 15 items and the practice section consists of 7 items. All items were single‐choice questions and adopted a 5‐point Likert scale, ranging from 1 (strongly unclear/disagree) to 5 (strongly clear/agree). A higher score indicates better knowledge, attitudes and practices.

Additionally, we graded the final scores. When the score is greater than or equal to 80% of the dimension score or the total score, we consider it an excellent score. When the score is < 60% of the dimension score or the total score, we consider it a negative score. When the score is between 60% and 80%, it is regarded as an intermediate score. For example, if the total score of the knowledge section is 35 points, when the score ≥ 35 × 80% = 28, it is considered excellent; when the score < 35 × 60% = 21, it is considered a negative score; when the score is between 21 and 28, it is regarded as an intermediate score.

### 4.3 Data Analysis

Statistical analysis was conducted using the statistical software package SPSS V.26.0 for Windows. Categorical variables are presented as frequencies and percentages. The normality of continuous variables was assessed using Kolmogorov–Smirnov tests (for sample sizes > 50) along with visual inspection of Q–Q plots and histograms. While the Kolmogorov–Smirnov tests indicated statistically significant departures from normality for all variables (*p* < 0.001), graphical analysis revealed that attitude, knowledge and total scores maintained approximately normal distributions, whereas practice scores exhibited non‐normal characteristics. Given the known robustness of parametric tests to mild violations of normality with large sample sizes (*n* = 404), independent samples *t*‐tests and ANOVA were used for normally distributed variables, with results reported as mean ± standard deviation. For non‐normally distributed practice scores, non‐parametric tests (Mann–Whitney *U* and Kruskal–Wallis) were employed. Relationships between knowledge, attitudes and practices were examined using Spearman's correlation coefficient due to the ordinal nature of the data. Statistically significant variables from univariate analyses were subsequently included in multiple regression models. A two‐tailed *p*‐value < 0.05 was considered statistically significant for all analyses.

### 4.4 Ethical and Institutional Approvals

This study obtained ethical approval (Approval No. 2020084) from the Medical Ethics Committee of West China Second Hospital of Sichuan University in May 2020.

## 5 Results

### 5.1 ICU Nurses' General Information

A total of 438 questionnaires were received. After eliminating 36 questionnaires due to over 30% of the information being incomplete, 404 valid ones were obtained. Among the participating nurses, 336 (83.2%) were female. A total of 212 (52.5%) nurses were under 30 years old. In terms of educational attainment, 314 (77.7%) held a bachelor's degree. Regarding ICU experience, 168 (41.6%) nurses had 5–10 years of experience in this field. There were 216 primary nurses (53.5%). As for the type of ICU, 222 (55.0%) nurses came from comprehensive ICUs and 322 (79.7%) nurses took care of children. Additionally, 384 (95.0%) nurses were from Grade III Class A hospitals. The detailed information is presented in Table 1.

**TABLE 1:** ICU nurses' general information (*n* = 404).

Variable	*n*	%
Gender		
Female	336	83.2
Male	68	16.8
Age group (years)		
< 30	212	52.5
31–40	174	43.1
> 41	18	4.5
Education level		
Diploma	72	17.8
Bachelor's degree	314	77.7
Master's degree or above	18	4.5
ICU experience (years)		
Less than 5	152	37.6
5–10	168	41.6
11–15	64	15.8
16–20	16	4.0
More than 20	4	1.0
Professional title		
Primary nurse	216	53.5
Intermediate/supervisor nurse	186	46.0
Senior	2	0.5
ICU classification		
Internal medicine ICU	174	43.1
Surgical ICU	8	2.0
General ICU	222	55.0
Nursing population		
Adult	46	11.4
Children	322	79.7
Mixed adult and children	36	8.9

Abbreviation: ICU, intensive care unit.

### 5.2 Scores of Each Dimension of the Questionnaire

In total, 64.4% of nurses were at the intermediate level (87–116 points) and 27.2% were at the excellent level (≥ 116 points). In the knowledge dimension, 70.8% of nurses obtained a negative score (< 21 points) and 26.2% were at the intermediate level (21–27 points). In the attitudes dimension, 49.0% of nurses were at the intermediate level (45–59 points) and 49.5% reached the excellent level (≥ 60 points). In the practices dimension, 22.8% of nurses had a negative score (< 21 points) and 39.1% were at the intermediate level (21–27 points). The specific information is detailed in Table 2.

**TABLE 2:** Scores of each dimension of the questionnaire (*n* = 404).

	Knowledge, *n* (%)	Attitudes, *n* (%)	Practices, *n* (%)	Total score, *n* (%)
Negative score	286 (70.8%)	6 (1.5%)	92 (22.8%)	34 (8.4%)
Intermediate score	106 (26.2%)	198 (49.0%)	158 (39.1%)	260 (64.4%)
Excellent score	12 (3.0%)	200 (49.5%)	154 (38.1%)	110 (27.2%)

In our study, all items were designed as single‐choice questions using a 5‐point Likert scale (ranging from 1 [strongly unclear/disagree] to 5 [strongly clear/agree]). We therefore calculated the mean scores with standard deviations for each item, ranked them based on their score values and presented the three highest‐ and lowest‐scoring items for each dimension in Table 3.

**TABLE 3:** The three items with the highest and lowest scores in each dimension.

Items	Score (mean ± SD)
Knowledge	
Do you know that critical care ultrasound technology can be used to improve the efficiency of nursing operations?	3.82 ± 0.889
Do you know that critical care ultrasound technology can be applied in the nursing field?	3.71 ± 0.863
Do you know if nurses can perform ultrasound operations independently?	2.52 ± 0.910
Do you know how to interpret the values shown on the ultrasound?	1.62 ± 0.710
Do you know how to interpret ultrasound images?	1.65 ± 0.676
Do you know the standards for clinical practice of ultrasound?	1.66 ± 0.738
Attitudes	
I believe that nurses' mastery of ultrasound technology can better ensure the safety of medical care.	4.42 ± 0.542
I believe that nurses need to master ultrasound technology in the rapid development of critical care medicine.	4.40 ± 0.575
I believe that critical care ultrasound will become a professional development direction for critical care nursing.	4.38 ± 0.579
I believe the use of critical care ultrasound will increase nurses' daily workload.	2.53 ± 1.164
I believe it is too difficult for nurses to master critical care ultrasound technology.	2.90 ± 0.959
I believe the use of critical care ultrasound will interfere with nurses' original clinical work.	3.28 ± 1.061
Practices	
I am paying attention to the scope of application of critical care ultrasound.	4.07 ± 0.630
I'm learning and practicing to interpret the meaning of the data shown in the ultrasound.	3.89 ± 0.805
I'm learning the key points of critical care ultrasound technology operation in my clinical work.	3.86 ± 0.835
I have received training related to critical care nursing ultrasound.	2.84 ± 1.363
I am deliberately practicing using the ultrasound machine.	3.45 ± 1.012
I am learning the theoretical knowledge related to critical care ultrasound technology.	3.75 ± 0.886

### 5.3 Factors Influencing the Different Dimensions of Univariate Analysis

Results of the univariate analysis revealed that the nursing population influenced respondents' performance in the knowledge dimension (*p* = 0.008), attitudes dimension (*p* < 0.01), practices dimension (*p* < 0.01) and the total score (*p* < 0.01), respectively. The ICU classification had significant influences on the knowledge dimension (*p* = 0.029). The education level had significant influences on the practices dimension (*p* = 0.003). The specific information is detailed in Table 4.

**TABLE 4:** The influence of ICU nurses' general information on different dimensions.

Variable	Knowledge		Attitudes		Practices		Total score	
	*F*/*t*‐test	*p*	*F*/*t*‐test	*p*	H	*p*	*F*/*t*‐test	*p*
Gender	1.853	0.065	−0.112	0.911	0.413	0.521	0.130	0.896
Age group	0.498	0.609	0.162	0.851	0.195	0.907	0.141	0.869
Education level	1.191	0.306	0.589	0.556	11.360	0.003*	0.436	0.647
ICU experience	2.109	0.081	0.312	0.870	0.894	0.925	0.309	0.872
Positional title	1.014	0.365	0.504	0.605	0.305	0.858	0.492	0.612
ICU classification	3.613	0.029*	1.687	0.188	1.781	0.411	1.374	0.256
Object of nursing	5.006	0.008**	9.792	0.000**	16.114	0.000**	11.520	0.000**
Hospital level	0.706	0.550	0.669	0.572	6.563	0.087	0.303	0.823

* *p* < 0.05.

** *p* < 0.01.

### 5.4 Correlation Analysis of Nurses' Knowledge, Attitudes and Practice Scores

The correlation analysis regarding nurses' knowledge, attitudes and practices was conducted. It was revealed that there was a significant positive correlation between knowledge and attitudes (*r* = 0.403, *p* < 0.01). Similarly, attitudes and practices also showed a positive correlation (*r* = 0.362, *p* < 0.01). Moreover, a positive correlation was observed between knowledge and practice as well (*r* = 0.434, *p* < 0.01).

### 5.5 Multivariate Linear Regression Analysis of General Data for Each Dimension

For ICU classification, General ICU served as the reference category (coded as 0,0,0), with Surgical ICU coded as (0,1,0) and Internal Medicine ICU coded as (0,0,1). For nursing population, adult patients were designated as the reference category (coded as 0,0,0), while children were coded as (0,1,0) and both adults and children were coded as (0,0,1).

The present study incorporated variables demonstrating significant associations in univariate analyses into multiple linear regression models (Table 5). The results indicated that ICU classification (surgical ICU vs. general ICU) significantly predicted lower scores across multiple domains: knowledge (*β* = −0.239, 95% CI [−10.661, −2.853], *p* < 0.01), attitudes (*β* = −0.163, 95% CI [−15.219, −1.322], *p* = 0.020) and total scores (*β* = −0.189, 95% CI [−29.499, −4.850], *p* < 0.01), with model explanatory power ranging from *R* ^2^ = 0.091 to 0.124. Similarly, when the nursing object was children compared to adults, significantly reduced scores were observed for knowledge (*β* = −0.285, 95% CI [−4.141, −1.435], *p* < 0.01), attitudes (*β* = −0.341, 95% CI [−8.379, −3.565], *p* < 0.01) and total scores (*β* = −0.233, 95% CI [−12.638, −1.989], *p* < 0.01). Notably, the nursing population, including both children and adults, demonstrated enhanced performance in the practice dimension (*β* = 0.285, 95% CI [2.707, 7.492], *p* < 0.01; *R* ^2^ = 0.077) and total scores (*β* = 0.182, 95% CI [0.710, 15.448], *p* < 0.01) relative to adult‐only cohorts.

**TABLE 5:** Multivariate linear regression analysis of general data for each dimension.

Variable	Knowledge		Attitudes		Practices		Total score	
	*β*	*t*‐test	*β*	*t*‐test	*β*	*t*‐test	*β*	*t*‐test
ICU classification								
Surgical	−0.239	−3.413**	−0.163	−2.347*	—	—	−0.189	−2.748**
Nursing population								
Children	−0.285	−4.065**	−0.341	−4.893**	—	—	−0.233	−2.709**
Mixed adult and children	—	—	—	—	0.285	4.203**	0.182	2.162*

* *p* < 0.05.

** *p* < 0.01.

## 6 Discussion

This study found that the majority of nurses had an intermediate total score on the questionnaire (64.4%). In the knowledge dimension, 70.8% of nurses had negative scores; in the attitude dimension, 49.0% of nurses had an intermediate attitude; in the practice dimension, 22.8% of nurses were unable to effectively carry out clinical practice. The study also indicated that factors such as the nursing population, ICU classification and education level had a significant impact on nurses' knowledge, attitudes and practices regarding CCUS, and their total scores. Moreover, there was a significant correlation between knowledge, attitudes and practices. Through multiple linear regression analysis, it was found that the nursing population and ICU classification were important factors influencing nurses' knowledge, attitudes and practices.

The knowledge, attitudes and practices (KAP) theory divides the process of human behaviour change into three steps, with knowledge serving as the foundational starting point, while beliefs and motives further drive behavioural transformation [27]. In our study, 70.8% of ICU nurses did not achieve satisfactory scores in critical care ultrasound knowledge. Difficulties in interpreting ultrasound images and data, along with unfamiliarity with operational standards, were key factors contributing to poor knowledge scores, consistent with the findings of Sun [23]. A study indicates that one of the risks associated with nurses' use of ultrasound stems from inexperienced or unskilled nurses potentially failing to accurately capture and interpret ultrasound images, which may result in misdiagnoses or missed diagnoses [28]. Several factors may explain these knowledge gaps. Firstly, continuous education programs and training opportunities in CCUS may be insufficient or inaccessible, leaving nurses unable to keep up with the latest advancements. A study revealed that formal training in bedside ultrasound is rare, with only 20% of institutions currently utilising this technology providing such training [29]. Secondly, the complexity of ICU work, where nurses often juggle multiple critical tasks, may leave limited time for in‐depth study of ultrasound. To address these challenges, hospitals and nursing educators should implement targeted interventions, such as offering short‐term online courses focused on image and data interpretation, and introducing collaborative programs with specialised ultrasound medical teams to enhance nurses' knowledge and skills.

Interestingly, while the knowledge level of ICU nurses was relatively low, nearly half (49.5%) achieved excellent scores in the attitudes dimension, suggesting that nurses recognise the importance and potential benefits of ultrasound in patient care despite their limited knowledge. This finding is consistent with surveys by other scholars: 89% of neonatologists reported a willingness to use ultrasound in clinical practice [30], and a nationwide UK survey revealed that 78.6% of advanced neonatal nurse practitioners are interested in receiving lung‐ultrasound training and implementing it in their units. From a theoretical perspective, according to the KAP theory, the positive attitude of nurses lays the foundation for the continuous development of ultrasound technology in the nursing field and can drive subsequent learning and skill improvement. The highest‐scoring attitude items reflected strong agreement with statements emphasising the importance of ultrasound technology for patient safety and professional development, including beliefs that ‘ultrasound technology can better ensure the safety of medical care’, ‘nurses need to master ultrasound technology in the rapid development of critical care medicine’ and ‘ultrasound will become a professional development direction for critical care nursing’. These results indicate a willingness among ICU nurses to adopt and integrate new technologies into their practice. However, the lowest‐scoring items, which were reverse‐scored to capture negative attitudes, revealed concerns about the practical challenges of implementing CCUS, such as the belief that ‘the use of CCUS will increase nurses' daily workload’, ‘it is too difficult for nurses to master CCUS technology’ and ‘the use of CCUS will interfere with nurses' busy routine work’. These low scores highlight nurses' concerns about the additional burden CCUS may place on their already demanding routines. Addressing these concerns by optimising the nurses' work processes (e.g., integrating critical care ultrasound technology into the nurses' work processes) and adding the positions of ultrasound nurses is crucial for the successful integration of CCUS into clinical practice.

In the practice dimension, 22.8% of nurses were unable to effectively carry out clinical practice, highlighting a significant gap in the practical application of critical care ultrasound (CCUS) among ICU nurses. A nationwide Canadian survey on pulmonary ultrasound applications revealed that 61% of neonatal nurses lacked any experience in utilising lung ultrasound [13]. This reveals a significant gap between China and other countries in the practical application of ultrasound technology within nursing care. The establishment of *Nursing Standards of Clinical Practice for Critical Care Ultrasonography* [24] might provide a clear framework to guide clinical nursing practice in China. The standards would help nurses select appropriate ultrasound examinations and nursing strategies when faced with clinical problems. Targeted nursing interventions could then be implemented based on assessment results, combined with clinical data and dynamically adjusted by evaluating the effectiveness of these interventions. Moreover, the shortage of ultrasound equipment remains a significant barrier to the widespread adoption and effective use of this technology [22]. To address this challenge, researchers have turned their focus to portable ultrasound devices as a viable solution. Portable ultrasound devices offer a practical solution to the cost and accessibility barriers of traditional systems, demonstrating significant potential in low‐resource environments [16]. In recent years, with the rapid development of AI technology, remote practical training can be carried out with the assistance of AI, and remote ultrasound diagnosis platforms can be developed [31, 32]. This enables medical institutions in remote areas or those with limited resources to obtain high‐quality ultrasound diagnosis support. One research study shows that the training effect of using a 5G remote interactive visual simulation and practical ultrasound system is consistent with that of traditional offline training [33]. The combination of ultrasound and AI may, to some extent, make up for the current deficiencies in the practical training, application and continuing education of ultrasound.

In terms of knowledge, attitudes and total scores, the performance of surgical and paediatric departments is relatively weaker. In surgical departments, the rapid turnover of patients and the clear aetiology of diseases may result in fewer opportunities to apply ultrasound technology, leading to lower levels of knowledge and less positive attitudes among nurses regarding critical care ultrasound. For paediatric departments, the development of critical care ultrasound technology has been relatively slower. For instance, while guidelines for adult ultrasound were published in 2015, paediatric ultrasound guidelines were not released until 2020 [34]. As a result, the introduction of critical care ultrasound technology in paediatric departments occurred later than in adult departments, and paediatric ICU nurses have had less time to familiarise themselves with it. This has led to lower scores in both knowledge and attitudes towards critical care ultrasound. However, the application of critical care ultrasound is particularly crucial in paediatric critical care, as it allows for safe procedures and rapid, convenient serial reassessments that improve diagnosis and monitoring [35, 36]. In terms of practice and total scores, departments that care for both adults and children scored higher than those that care exclusively for adults. This may be attributed to better medical and training resources in comprehensive hospitals.

## 7 Limitations

This study has several limitations. First, the use of convenience sampling may limit the generalisability of the findings. Second, the cross‐sectional design precludes causal inferences. Longitudinal studies are needed to assess the impact of training interventions over time. Third, the self‐reported nature of the questionnaire data may introduce potential biases, particularly social desirability bias (e.g., nurses may overreport positive attitudes or practices). While we have rigorously controlled for measurable confounding factors through multivariate adjustment, residual confounding may persist due to unmeasured variables such as individual learning capacity or personal social resources. Further refinement of the measurement tools and inclusion of qualitative data, such as in‐depth interviews or focus groups, could offer a more comprehensive understanding.

## 8 Recommendations or Implications for Practice and/or Further Research

The study on the knowledge, attitudes and practices of ICU nurses regarding critical care ultrasound (CCUS) in Southwestern China provides valuable insights for nursing practice. The findings indicate that ICU nurses generally hold positive attitudes toward CCUS, recognising its potential to enhance patient care and improve clinical outcomes. However, the study also highlights a significant gap in theoretical knowledge and practical application of CCUS among nurses, which limits its full utilisation in critical care settings. Furthermore, the positive attitudes identified in the study suggest that ICU nurses are motivated to learn and apply CCUS, which can be leveraged to promote a culture of continuous learning and innovation in critical care units. By fostering interdisciplinary collaboration between nurses, physicians and sonographers, healthcare institutions can enhance the overall quality of care and patient outcomes.

Nurse clinicians and educators can use this evidence to design targeted training programmes aimed at improving ICU nurses' theoretical understanding and technical proficiency in CCUS. By addressing these knowledge gaps, nurses can better integrate CCUS into their daily practice, leading to more accurate assessments and timely interventions for critically ill patients. Additionally, nurse managers should prioritise creating supportive environments that encourage the adoption of CCUS, such as providing access to training resources, equipment and mentorship from experienced practitioners.

## 9 Conclusion

ICU nurses hold a positive attitude toward critical care ultrasound technology, generally believing that it can assist their work and improve patient outcomes. However, the current practical application of critical care ultrasound by ICU nurses is insufficient, particularly due to a lack of theoretical knowledge related to the technology.

## Ethics Statement

This study obtained ethical approval (Approval No. 2020084) from the Medical Ethics Committee of West China Second Hospital of Sichuan University in May 2020.

## Consent

Informed consent was obtained from all nurses after explaining the aim of the study prior to completing the questionnaire. Participation was voluntary, anonymous and respondents were informed of their right to withdraw at any time.

## Conflicts of Interest

The authors declare no conflicts of interest.

## Data Availability

The data that support the findings of this study are available from the corresponding author upon reasonable request.
